# Mechanism of C-N bonds formation in electrocatalytic urea production revealed by ab initio molecular dynamics simulation

**DOI:** 10.1038/s41467-022-33258-0

**Published:** 2022-09-17

**Authors:** Xin Liu, Yan Jiao, Yao Zheng, Mietek Jaroniec, Shi-Zhang Qiao

**Affiliations:** 1 School of Chemical Engineering and Advanced Materials, The University of Adelaide, Adelaide, SA 5005 Australia; 2 Centre for Materials in Energy and Catalysis, The University of Adelaide, Adelaide, SA 5005 Australia; 3 Department of Chemistry and Biochemistry & Advanced Materials and Liquid Crystal Institute, Kent State University, Kent, OH 44242 USA

**Keywords:** Density functional theory, Computational chemistry, Heterogeneous catalysis, Electrochemistry

## Abstract

Electrosynthesis of urea from CO_2_ and NO_X_ provides an exceptional opportunity for human society, given the increasingly available renewable energy. Urea electrosynthesis is challenging. In order to raise the overall electrosynthesis efficiency, the most critical reaction step for such electrosynthesis, C-N coupling, needs to be significantly improved. The C-N coupling can only happen at a narrow potential window, generally in the low overpotential region, and a fundamental understanding of the C-N coupling is needed for further development of this strategy. In this regard, we perform ab initio Molecular Dynamics simulations to reveal the origin of C-N coupling under a small electrode potential window with both the dynamic nature of water as a solvent, and the electrode potentials considered. We explore the key reaction networks for urea formation on Cu(100) surface in neutral electrolytes. Our work shows excellent agreement with experimentally observed selectivity under different potentials on the Cu electrode. We discover that the ^*^NH and ^*^CO are the key precursors for C-N bonds formation at low overpotential, while at high overpotential the C-N coupling occurs between adsorbed ^*^NH and solvated CO. These insights provide vital information for future spectroscopic measurements and enable us to design new electrochemical systems for more value-added chemicals.

## Abstract (web summary)

Urea electrosyntehsis from CO2 and NOx is a challenging reaction that is becoming increasingly important. This work uses ab initio molecular dynamics simulations to reveal the origin of C-N coupling mechanisms and reaction networks in urea synthesis.

## Introduction

The importance of artificial nitrogen fertilizer, *i.e*. urea, production is underpinned by the fact that these fertilizers led to about 30-50% of the crop yield increase assured food for almost half of humanity^1^. However, the associated processes –feedstock ammonia synthesis through Haber-Bosch, and urea synthesis – greatly rely on fossil fuel resources, both as raw materials and energy sources, which is highly unsustainable with heavy CO_2_ emission^2^. Another critical problem associated with artificial nitrogen fertilizer is the accumulation of reactive nitrogen, which accounts for algal blooms and leads to a decline in the quality of surface and ground waters^3^. Using renewable electricity to electrochemically convert CO_2_ and reactive nitrogen to urea could solve the two issues at the same time, enabling decarbonization of the nitrogen fertilizers production industry and assuring better sustainability as well as on-the-site and on-demand production of nitrogen fertilizers^4–7^.

The most investigated nitrogen source for electrochemical urea production was ammonia and di-nitrogen; however, this incurs additional process and cost for ammonia collection and purification^2,8–15^, and shows low Faradic efficiency (FE)^16^. An alternative nitrogen source is nitrate/nitrite ions (NO_3_^-^/NO_2_^-^), which could improve the FE for urea production (35% on copper; over 40% on TiO_2_-based catalysts)^17–23^. An additional benefit of using nitrate/nitrite ions as the nitrogen source for urea production is their sourcing from wastewater, which contributes to reducing the excess of reactive nitrogen in the ecosystem.

To further improve the efficiency of urea synthesis using nitrate/nitrite ions as feedstock, the reaction mechanism for direct electrocatalytic urea production needs a better understanding, especially the C-N coupling step. C-N coupling can only happen at a narrow potential window, generally in the low overpotential region^16–21,24^. So far, the exact reaction pathways for urea formation at this narrow potential windows remain an open question^16,19,24^. Previous works either focus on synthetic organic chemistry without consideration of electrode potentials^25,26^, or C-N formation from different reactants *e.g*. via ^*^NCON^*^ formation through ^*^N_2_ and ^*^CO coupling^16^, and via NH_3_ nucleophilic attack on ketene intermediate from ammonia and CO_(g)_^24^. Therefore, the mechanisms for C-N formation from NO_X_ and CO_2_ at specific potentials have remained elusive.

Such C-N coupling mechanism can be investigated by first principle-based molecular modelling because this methodology provides atomic-level insights into electrocatalytic reactions^27^. However, earlier attempts usually adopted vacuum models; the effect of solvent and applied potentials are ignored during the calculation and were considered at a later stage by mathematical methods^28,29^. Several recent efforts involved implicit or hybrid implicit/explicit models with a few water molecules; however, these calculations might lead to inconsistent kinetic barrier values^30–32^. To better consider the dynamic nature of water and thermodynamics ensemble effect, as well as the impact from hydrogen bonds and electrode potentials, ab initio Molecular Dynamics (AIMD) simulations should be adopted and give more reliable results^33–38^.

In this work, we performed AIMD simulations to explore the reaction mechanisms of the key steps for simultaneous electrochemical reduction of CO_2_ and NO_3_^–^/NO_2_^–^ towards urea formation on the Cu(100) surface as a model catalyst. With the solvent and applied potential effects taken into considerations, a potential-dependent mechanism was found to account for the selective formation of urea at the appropriate potential window. A two-step coupling between ^*^NH and ^*^CO constitutes the C-N bond formation at low overpotential region as evidenced by careful examining different coupling intermediates and competing protonation reactions. At higher overpotential, the selectivity toward urea formation is reduced due to the enhancement of competing reactions; also, this process proceeds via different C-N coupling reaction mechanism involving desorbed CO. Our work provides atomic mechanism of urea formation and shows excellent agreement with key experimental observation on the Cu electrode^19^. This work will stimulate the development of electrochemical methods utilizing CO_2_ and NO_3_^–^/NO_2_^–^ to synthesize urea or more value-added compounds with C-N bond formation at ambient conditions, as well as the future design of relevant catalyst materials.

## Results

### Pathways toward ammonia at low overpotential

**Fig. 1:**
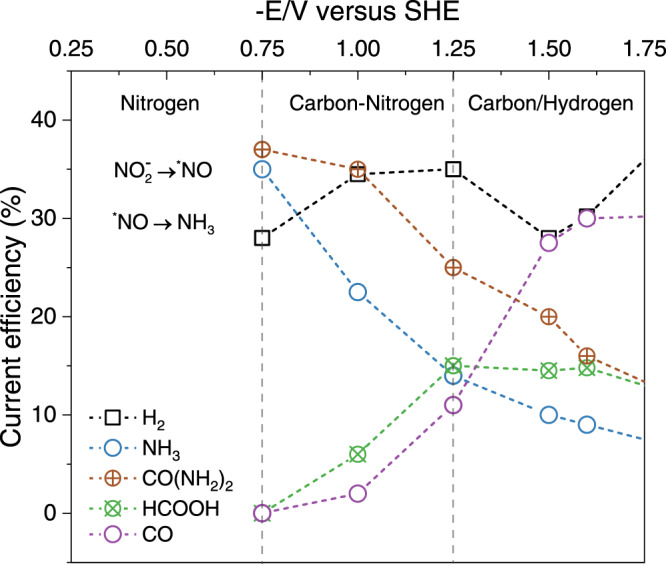
Co-electroreduction of CO_2 _ and NO_2_^−^ on copper electrode. Experimentally determined current efficiency for the major reduction products from CO_2_ and NO_2_^**–**^ at a Cu loaded gas-diffusion electrode as a function of the electrode potential, reproduced with permission from the Shibata et al^19^. article. Copyright 1998, Elsevier.

As a representative simultaneous electroreduction of CO_2_ and nitrite ions, the current efficiency of main products as a function of potential measured by Shibata *et al*. with a Cu gas-diffusion electrode, is reproduced and shown in Fig. 1^19^. Ammonia (NH_3_) and urea (CO(NH_2_)_2_) are the dominant products at -0.75 V *vs* SHE (standard hydrogen electrode), which are produced by nitrite reduction and electrochemical C-N coupling, respectively. At more negative potentials, the formation of carbon monoxide (CO) and formic acid (HCOOH) via CO_2_ reduction increases monotonically while production of NH_3_ and CO(NH_2_)_2_ continuously decreases.

We first constructed and equilibrated the electrochemical Cu (100)/water interface with various reaction intermediates. The performed AIMD simulations of the as-constructed interface were used to obtain the temperature and potential energy profiles shown in Supplementary Fig. S1. Information on the calculation of the electrode potential at these interfaces, and detailed workflow are provided in the section devoted to the Determination of Electrode Potential and Supplementary Fig. S2. The work functions used to calculate electrode potential are summarized in Supplementary Tables S1-2^39^. We found that our calculated electrode potential values (-0.85 ~ -0.59 V *vs* SHE) are in accordance with the experimental value (-0.73 V *vs* SHE) for the potential of zero charge of single crystal Cu(100) under neutral environment by Łukomska and Sobkowski^40^.

**Fig. 2:**
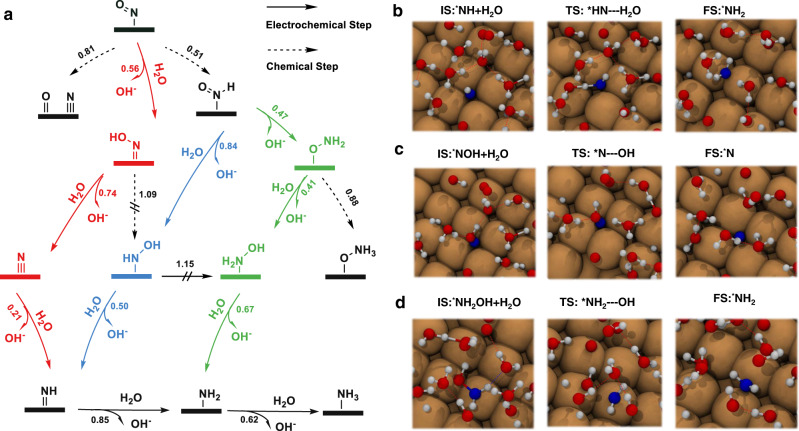
Reaction pathways and snapshots for *NO reduction to NH3. **a** Schematic diagram of kinetically preferred reaction pathway from ^*^NO to ammonia (NH_3_) at ~ −0.75 V *vs* SHE. Both chemical and electrochemical steps are considered. Chemical reaction steps are indicated by dashed lines, whereas electrochemical steps are indicated by solid lines. Steps with a barrier higher than 1.0 eV are marked with a doubled bar line (||) and considered as precluded (blocked) steps at 300 K. All kinetic barrier values are reported in eV. Three different pathways are marked in red (i), blue (ii) and green (iii), while shared steps are marked in black. **b-d** Snapshots of reactive trajectories for three possible rate-determining steps towards ammonia formation. **b** Protonation of ^*^NH to ^*^NH_2_; **c**, dehydroxylation of ^*^NOH to ^*^N; **d**, dehydroxylation of ^*^NH_2_OH to ^*^NH_2_. Color code: hydrogen in white, oxygen in red, copper in brown, carbon in black, nitrogen in blue, hydrogen bonds in red dashed lines.

Figure 2a depicts the most relevant reaction pathways starting from ^*^NO, as well as the reaction barrier values for each step using the AIMD-derived thermodynamic integration method (as shown in Supplementary Table S3). A detailed discussion on the accuracy of the estimation of barriers could be found in Supplementary Figs. S3–S5. ^*^NO could be reduced to either ^*^NOH or ^*^HNO; in the meantime, it also could dissociate into atomic nitrogen and oxygen^41^. We found that the formation of ^*^HNO is via a chemical step (*i.e*., without the direct involvement of proton and electron transfer) preceded by surface hydrogenation. Whereas ^*^NOH is formed through an electrochemical reduction step (*i.e*., with the involvement of proton and electron transfer) with water molecule as the proton source; this is similar to the case of CO^*^ in CO_2_/CO reduction at a small overpotential^34,42^. Dissociation of ^*^NO to ^*^N and ^*^O with free energy barrier 0.81 eV is kinetically less favorable than protonation to ^*^HNO (0.51 eV) or to ^*^NOH (0.56 eV). For further reduction steps of ^*^HNO or ^*^NOH, various intermediates were involved in different pathways. We identified three kinetic favorable pathways for NO^*^ reduction toward ammonia: i) ^*^NO → ^*^NOH → ^*^N → ^*^NH → ^*^NH_2_ → ^*^NH_3_ (red); ii)^*^NO → ^*^HNO → ^*^NHOH → ^*^NH → ^*^NH_2_ → ^*^NH_3_ (blue); iii)^*^NO → ^*^HNO → ^*^ONH_2_ → ^*^NH_2_OH → ^*^NH_2_ → ^*^NH_3_ (green). Both i) and ii) pathways end with the formation of ^*^NH, which is gradually protonated to NH_3_. For pathway i) the reduction starts with ^*^NOH with the formation of ^*^N + H_2_O (red in Fig. 2a). For pathway ii), the ^*^NH formation occurs through ^*^HNO and ^*^NHOH (blue). Pathway iii) shares the same precursor as ii) – ^*^HNO and bases on the formation of ^*^NH_2_ (green).

There are many possible rate-limiting steps for these three pathways. For pathways i) and ii), as shown in Fig. 2b, protonation of ^*^NH to ^*^NH_2_ via an electrochemical step is the rate-determining step with a barrier value of 0.85 eV. The dehydroxylation of ^*^NOH as displayed in Fig. 2c could also be considered as the rate-determining step for pathway i), because of a relatively high barrier value of 0.74 eV, which is comparable with that of ^*^NH protonation to ^*^NH_2_ considering the estimated errors.

For pathway ii), protonation of ^*^HNO and dehydroxylation of HNOH^*^ via electrochemical mechanism are facile with barriers of 0.84 eV and 0.50 eV, respectively. While for pathway iii), the successive formation of ^*^ONH_2_ and ^*^NH_2_OH through an electrochemical step is facile as indicated by barrier values of 0.47 eV and 0.41 eV, respectively. Thus, the dehydroxylation of ^*^NH_2_OH to ^*^NH_2_ has a free energy change of 0.67 eV (Fig. 2d) and is regarded as the rate-determining step. In summary, although three different pathways possess various intermediates, the kinetic barriers toward common product ammonia are very close (0.67-0.85 eV).

Since Cu(100) surface has been identified as the major active facet under similar reduction reaction conditions, we compare our results from AIMD simulations with experiments conducted on copper-based catalysts^43^. The most significant agreement between previous experiments and our theoretical prediction is that ammonia is the major product but not N_2_O or N_2_ in neutral electrolyte^17,44,45^. This can be explained by the ultra-low barrier of ^*^N protonation to NH^*^ (0.21 eV), and such a low barrier inhibits ^*^N coupling with another ^*^N or ^*^NO to form N_2_ and N_2_O, respectively. We found that in pathway iii), ^*^NH_2_OH is a key intermediate toward the formation of ammonia. These results are in accordance with the recent report that ^*^NH_2_OH was detected as an intermediate during nitrate reduction to ammonia on copper-based materials by online differential electrochemical mass spectrometry^45^. However, Pérez-Gallent et al. found that hydroxylamine (NH_2_OH) is the final product for nitrate reduction on Cu(100) in alkaline media^46^. One possible explanation is that this pathway is sensitive to pH as well as the applied electrode potential. To validate pathways i) and ii), determination of intermediates like ^*^NH or ^*^NHOH via spectroscopy-based characterization is still challenging, and we believe that a combination of electrochemical measurements coupled with in situ characterization techniques as well as advanced atomic simulation methods would benefit determination of the complex reaction mechanisms^47,48^.

### The first C-N bond formation at low overpotential

The previous section proved that reduction of ^*^NO toward ammonia is facile at about -0.75 V *vs* SHE, indicating that there should be considerate amounts of various related intermediates on the catalyst surface. At the same time, if CO_2_ is also involved, it has been reported that ethylene starts to form on Cu(100) surface from -0.81 V *vs* SHE in neutral solutions^49^. In view of these two points, there could be a potential window, within which dimerization of ^*^CO is not dominant (or even does not begin) whereas nitrogen-containing intermediates are accumulated on the surface because of the rapid reduction of ^*^NO^46^. Keeping this in mind, we hypothesize that within this potential window, ^*^CO could couple with nitrogen intermediates and C-N would be formed. This hypothesis is in accordance with the previous experimental reports on the production of urea (two amide bonds) by simultaneous reduction of carbon dioxide and nitrite or nitrate ions^17–21,23^. However, the detailed reaction mechanisms of urea formation are still absent.

**Fig. 3:**
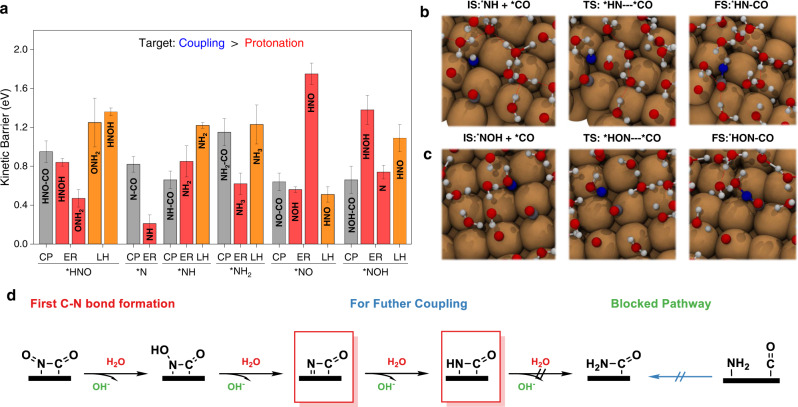
The first C-N bond formation. **a** comparison of the coupling energy barrier of various nitrogenous intermediates with ^*^CO and protonation for ^*^NO, ^*^NOH, ^*^NH, ^*^HNO, ^*^N and ^*^NH_2_. CP stands for coupling, ER for Eley-Rideal mechanism, and LH for Langmuir-Hinshelwood mechanism. Snapshots of reactive trajectories for the ^*^CO coupling with **b**, ^*^NO, **c**, ^*^NOH. **d** Schematic diagram for the formation of the first C-N bond as well as subsequent protonation.

The first step is to investigate the mechanisms of the coupling intermediates to form C-N bond. For carbonaceous (C-) intermediates, ^*^CO was chosen because it is a key intermediate for CO_2_/CO reduction, as well as it’s abundant on the Cu(100) surface at this potential due to a sluggish protonation of ^*^CO^28,29,35,49,50^. For the nitrogenous intermediates (N-), a number of candidate intermediates relevant to the NO-to-NH_3_ pathway need to be considered. Also, the protonation of N- intermediates via Eley-Rideal (ER; proton from solution) or Langmuir-Hinshelwood (LH; proton from surface adsorption) mechanism could compete with coupling reactions with ^*^CO. After considering all possible reaction pathways, including C-N coupling and protonation, the reaction barrier values are summarized in Fig. 3a (details are given in Supplementary Table S3 and S4). These two panels indicate that for ^*^NOH and ^*^NH, the C-N coupling is more facile than the alternative protonation via LH or ER mechanism (Fig. 3a). The snapshots of reactive trajectories for these two coupling steps are displayed in Fig. 3b-c. The calculated reaction barriers are both 0.66 eV, demonstrating relatively fast kinetics. In comparison, the coupling of ^*^CO with ^*^HNO, ^*^N and ^*^NH_2_ is kinetically precluded (blocked) at room temperature and protonation reactions are favored. We noticed that although the coupling of ^*^CO and ^*^NH_2_ was previously claimed as the rate-determining step of ammonia formation, our calculated reaction barrier is 1.15 eV, which is less favorable than protonation to ammonia via ER mechanism and could be regarded as blocked at 300 K^18,22,23^.

For ^*^NO, considering the relatively close value of protonation (0.56 eV for ER to ^*^NOH and 0.51 eV for LH to ^*^HNO) and C-N coupling barrier (0.64 eV) and the estimated error of the adopted methodology as shown in Table S4, we investigated three possible C-N intermediates, namely, ^*^NO-CO, ^*^NOH-CO, and ^*^NH-CO for the following steps towards urea formation. Similar to previous considerations, we shall compare the kinetic barrier of protonation as well as further coupling with the second ^*^CO of these C-N intermediates. Starting from ^*^NO-CO, it needs four successive reduction steps toward ^*^NH_2_-CO, as evidenced by the detailed results provided in Table S5. As shown in Fig. 3d, protonation of ^*^NO-CO and ^*^NOH-CO is very facile with reaction barriers of 0.12 and 0.36 eV, respectively. However, both ^*^N-CO (to ^*^NH-CO) and the following ^*^NH-CO (to ^*^NH_2_-CO) are hard to be reduced at this potential due to the large reaction barrier. Furthermore, ^*^NH_2_-CO could not be formed under this scenario since the direct coupling of ^*^NH_2_ and ^*^CO is excluded as discussed previously. In conclusion, only ^*^N-CO and ^*^NH-CO could be considered for further coupling at this stage.

### The second C-N bond formation at low overpotential

**Fig. 4:**
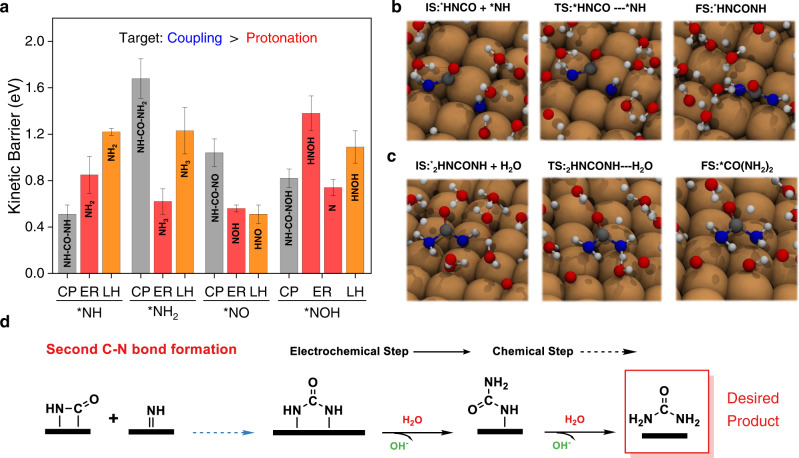
The second C-N bond formation. **a** Comparison of the coupling of various nitrogenous intermediates with ^*^CO-NH and protonation. Snapshots of reactive trajectories for **b** ^*^NHCO coupling with ^*^NH, **c** ^*^NH_2_CONH protonation to urea. **d** Schematic diagram for the formation of the second C-N bond as well as subsequent protonation.

To form urea, another C-N bond needs to be formed, and further couplings of the previously identified ^*^N-CO and ^*^NH-CO with various N-intermediates (^*^NO, ^*^NOH, ^*^NH and ^*^NH_2_) to form the second C-N bond should be explored. We excluded ^*^N and ^*^HNO because previous discussions have demonstrated that the relatively low barrier of protonation to form ^*^NH and ^*^ONH_2_, respectively. As such, ^*^N/^*^HNO couplings with ^*^N-CO or ^*^NH-CO are difficult to proceed with, similar to in the case of the first C-N bond formation. As for^*^N-CO intermediate, as shown in Supplementary Fig. S6, the coupling with N-intermediates to form the second C-N bond is less favorable (all kinetic barrier values are higher than 0.93 eV) than protonation for all of the possible precursors (^*^NO, ^*^NOH, ^*^NH, and ^*^NH_2_). Therefore, ^*^N-CO is not reactive towards urea production. For ^*^NH-CO, the coupling barriers to form the second C-N bond are summarized in Fig. 4a, and ^*^NH is the only intermediate that could be coupled with ^*^CO-NH (more details could be found in Supplementary Table S5). The associated barrier value is 0.51 eV for coupling ^*^NH with ^*^NH-CO to form ^*^NH-CO-NH (Supplementary Table S6), which is also lower than ^*^NH protonation (0.85 eV), and favors coupling instead of protonation. While for ^*^NO, ^*^NOH, and ^*^NH_2_, the barriers are relatively large and less facile than the corresponding protonation of these N-intermediates as shown in Fig.4a. Once ^*^NH-CO-NH is formed as displayed in Fig. 4b after two consecutive coupling steps between ^*^CO and ^*^NH, the key intermediate experiences further protonation to urea ((NH_2_)_2_CO). Protonation of ^*^NH-CO-NH is predicted to be facile with two barriers of 0.52 eV and 0.50 eV via the ER mechanism (Supplementary Table S7), and the step is shown in Fig. 4c.

To summarize, we found that at about -0.75 vs SHE, the kinetically favorable pathway from ^*^NH and ^*^CO to urea is ^*^CO + 2^*^NH → ^*^NH-CO + ^*^NH → ^*^CO(NH)_2_ → ^*^NH_2_-CO-NH → (NH_2_)_2_CO. Each step of the C-N bond formation process has a lower kinetic barrier than protonation of ^*^NH^*^ to ^*^NH_2_ (0.85 eV), indicating the selectivity to urea formation is higher than, or at least comparable to, ammonia formation at such a potential. This prediction is in accordance with experimental reports by Shibata et al that the current efficiency for urea and ammonia is 37% and 35%, respectively as shown in Fig. 1^19^. However, it is hard to determine, which is the rate determining step and several steps might be regarded as the rate destemming steps on the pathway to urea due to the comparable values of kinetic barriers, which are actually within the range of intrinsic DFT (Density functional theory) calculation errors as well as the error range of our computations^38^. To validate the C-N formation mechanism we proposed, based on the AIMD simulations, that the key intermediates like ^*^CO-NH and ^*^CO(NH)_2_ are subject to future experimental measurements by advanced characterization techniques on well-defined systems^46,49,51^.

### Decreased urea and ammonia production at high overpotential

As shown in Fig. 1, the selectivity toward ammonia and urea is highly potential dependent. From -0.75 V to -1.5 V *vs* SHE, the current efficiency toward ammonia and urea formation continues to decrease, while CO_(g)_ and HCOOH production are accelerated. To reveal the origins of this behavior, we introduced one lithium atom into the system and thus adjusted the potential to about -1.5 V *vs* SHE and employed AIMD simulations to explore the kinetics of related reaction pathways (results are summarized in Supplementary Table S8).

**Fig. 5:**
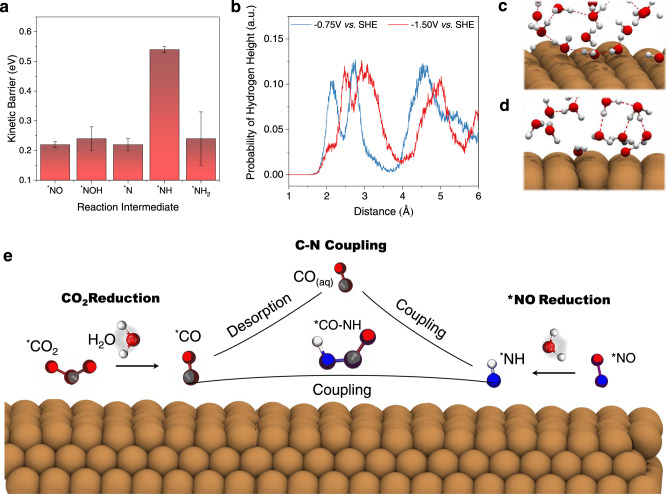
Reaction mechanisms at about -1.5 V vs SHE. **a** Kinetic barriers of ^*^NO reduction toward ammonia (NH_3_) formation at ~ -1.5 V *vs* SHE on the Cu(100) surface. **b** Probability of distribution of hydrogen height along direction normal to the Cu(100) surface. Snapshots of the interface at about **c** −0.75 V and **d** −1.5 V vs SHE (intermediates are removed for clarity). **e** Schematic diagrams for two different routes of C-N coupling: surface-mediated coupling of ^*^NH with ^*^CO or ^*^NH direct coupling with CO_(aq)_.

We first discuss the ^*^NO reduction to ^*^NH_3_ pathway. The results at this potential shown in Fig. 5a indicate that reduction of ^*^NO is facile due to low kinetic barriers of each step. We found that ammonia is the only dominant product for ^*^NO reduction, and the most kinetically favorable pathway is ^*^NO → ^*^NOH → ^*^N → ^*^NH → ^*^NH_2_ → ^*^NH_3_. What’s more, all steps prefer ER mechanism and the barriers for almost all steps are lower than those for the scenario at low overpotential (more details could be found in Supplementary Table S8). This finding is in accordance with previous reports that larger overpotential would reduce the barrier of protonation via electrochemical pathways^31,42,52^. We also found that at these two distinct potentials, the interface structure is different. The distribution of hydrogen atoms for water molecules shows different behavior as presented in Fig. 5b. At a very negative potential of -1.5 V *vs* SHE, the adsorbed water molecules tend to decrease compared with those for -0.75 V *vs* SHE. Snapshots in Figs. 5c and 5d indicate that at higher overpotential applied, hydrogen atoms of water molecules in the outer Helmholtz layer tend to point toward the negatively charged surface. All these data indicate that as the applied potential decreases, the structure of Helmholtz layer changes, which accounts for the difference in protonation kinetics of reaction intermediates. To summarize, more negative potential enhances the reduction of ^*^NO to ammonia. Therefore, the experimental observation indicating the reduced current efficiency toward ammonia formation should be attributed to the competing reaction.

We then explored the C-N coupling reaction via ^*^NH and surface adsorbed ^*^CO at the same potential because this is the key step for ammonia formation at -0.75 V *vs* SHE. However, the calculated reaction barrier for this surface coupling reaction step is 1.25 eV, indicating that this pathway is blocked due to a large reaction barrier at room temperature. Hence, there should be a different pathway for the C-N bond formation and urea production at such high overpotential. According to Fig. 1, as the applied electrode potential decreases, the release of gaseous hydrogen and carbon monoxide increase, which indicate the enhancement of competing hydrogen evolution and ^*^CO desorption. This could be attributed to the fact that more negative potential benefits hydrogen adsorption as Cheng et al claimed^34^. We found that the surface adsorbed ^*^NH is capable of coupling with non-adsorbed CO to form ^*^CO-NH with a barrier of 0.62 eV. A similar mechanism was previously proposed for CO-CHO coupling at large overpotential in CO_(2)_ reduction^37^. These two mechanisms are shown in Fig. 5e; namely, adsorbed ^*^CO participates in coupling with the surface adsorbed ^*^NH at a relatively positive potential, while at a very negative potential CO molecule in solution originating from ^*^CO desorption could directly react with ^*^NH. Hence there is still urea production at -1.5 V *vs* SHE as shown in Fig. 1.

Besides ^*^NO reduction and C-N coupling, CO_2_ reduction and hydrogen evolution reactions also occur at the same time under the high overpotential window, leading to a wide spectrum of products including CO_(g)_, HCOOH and H_2_ other than ammonia and urea. It is believed that these competing reactions are indispensable for explaining the experimental observation of decreased current efficiency toward urea and ammonia formation. Since the production of carbon monoxide significantly increases, it is rational to infer that reduction of CO_2_ to CO/HCOOH is accelerated and these reactions would compete with ^*^NO reduction for proton source from water molecule at the interface. Therefore, we inferred that production of ammonia slows down due to the less favorable kinetics than CO_2_ reduction. The surface coverage of associated ^*^NH would decrease and affect both ammonia and urea formation because of its crucial role it in the reaction network. If the potential continues to become more cathodic, hydrogen evolution reaction would dominate due to a dramatically boosted the Volmer step (H^+^ + e^-^ →^*^H)^19,35^. Therefore, H_2(g)_ eventually becomes the only final product, similarly as in the case of sole CO_2_ reduction^50^.

## Discussion

By adopting ab initio Molecular Dynamics simulation, which could describe the electrode potential and dynamic nature of water in the electrolyte, we identified the reaction pathways for urea and ammonia production on Cu (100) surface at the neutral solution. After exploring various combinations of coupling intermediates, we elucidated the role of ^*^NH and ^*^CO as the critical surface intermediates for C-N coupling along the urea pathway under low overpotentials. At higher overpotentials, C-N coupling proceeds with a different mechanism that is impacted by competitive CO_(2)_ reduction, which leads to the narrow potential window for urea production. Our modelling results successfully explain the experimentally observed activity/selectivity for simultaneous reduction of CO_(2)_ and nitrogen source (nitrite/nitrate), and demonstrate that the knowledge of the electrode potential and the dynamic water structure at the same time is critical in modelling potential dependent electrocatalytic reactions. These insights in reaction mechanisms enable us to design new electrochemical systems, provide vital information for future spectroscopic measurements, and pave the way for catalyst materials to synthesize more value-added chemicals to meet future energy and environmental challenges. A new opportunity emerges: using electrolysis systems as a platform to produce a broader range of complex compounds other than simple carbon species or nitrogen-containing species to cover more aspects of the modern chemical production supply chain^38,53^.

## Methods

### Considerations of the catalyst, pH, electrode potential and starting reactants

Copper is selected as the model catalyst since it was found to display a high selectivity of urea^19^. Here, we simulate the Cu(100)/water interface using 32 explicit water molecules on 3×4×3 Cu(100) surface slab, because Cu(100) proved to be a dominant surface for polycrystalline copper when a considerate reductive potential is applied^43^. The size of chosen system was based on previous report on CO_2_ reduction, where also three layers of copper and around thirty water molecules were used^33,34^. For the selection of solution pH values, we only focus on the neutral condition. The reason is that in acidic solutions, NO_3_^-^ or NO_2_^-^ are involved in competing homogeneous side reactions. In alkaline solutions, surface CO_2_ reduction is suppressed due to the undesired carbonates generation^54^. At the same time, the available experimental data on the urea generation from CO_2_ and NO_3_^-^/NO_2_^-^ were conducted in neutral solutions, which could serve as a key benchmark for our computation study^19^.

Two electrode potential windows were investigated according to Fig. 1. The first potential of interest is -0.75 V *vs* SHE where the current efficiency toward urea formation reached the maximum, which we refer to as the low overpotential. When electrode potential becomes more negative, the urea and ammonia production efficiency decrease monotonously which is accompanied with an increase in CO production. The high overpotential region around -1.5 V *vs* SHE is modelled by introducing one lithium atom into the Cu(100)/water interface.

Another consideration is the starting reactants that would simplify the problem allowing focus on the critical aspects of the reaction networks. The formation of urea needs both nitrogen and carbon source. In CO_2_ electrocatalysis, any deeply reduced product must be formed through ^*^CO; hence, ^*^CO was chosen as the starting carbon intermediate. As for the nitrogen source, both NO_3_^-^ and NO_2_^-^ are further reduced to ^*^NO when the applied potential is relatively negative as the case in the current work^46,54^. Therefore, we adopt ^*^NO and ^*^CO as the starting reactants to investigate the crucial aspects of the reaction networks and explore the C-N bond formation mechanisms.

### Density functional theory computations

DFT method was employed for all calculations using the Vienna ab initio Simulation Package (VASP)^55–58^. The projector-augmented-wave pseudo-potential was utilized to treat the core electrons, while the Perdew-Burke-Ernzerhof (PBE) exchange-correlation functional of the generalized gradient approximation (GGA) was used for describing the electron interactions^59^. The van der Waals interactions were described using the empirical correction in the Grimme’s scheme^55,60^.

We carried out AIMD simulations to equilibrate the system at 300 K for over 30 ps, with a time step of 1 fs. Based on the well-equilibrated interface, the reaction barriers were estimated by thermodynamic integration based on Blue Moon ensemble method^61^. Both electrochemical (Eley-Rideal) and chemical (Langmuir-Hinshelwood) reaction mechanisms were considered for intermediate hydrogenation. A more detailed description of the set-up of AIMD simulations and electrode potential calculations could be found in the Supporting Information section devoted to the computational method.

## Supplementary information

Supplementary Information

## Data Availability

Data supporting the reported findings are available in the article and Supplementary Information and Source Data file. All trajectories are deposited in the open database of Materials Cloud (10.24435/materialscloud:8t-6e). Source data are provided with this paper.

## Source data

Source Data
