# Supplementary material for: Mechanism of C-N bonds formation in electrocatalytic urea production revealed by ab initio molecular dynamics simulation
Source: Nat Commun. 2022 Sep 17;13:5471. doi: 10.1038/s41467-022-33258-0 (PMC9482648; doi:10.1038/s41467-022-33258-0)
Supplement: Supplementary file 1 — Supplementary Information [file 41467_2022_33258_MOESM1_ESM.pdf]

Supporting Information

Mechanism of C-N Bonds Formation in Electrocatalytic Urea Production Revealed by *ab initio* Molecular Dynamics Simulation

Xin Liu,[1,2] Yan Jiao,[*,1,2] Yao Zheng,[1,2] Mietek Jaroniec,[3] Shi-Zhang Qiao[*,1,2]

[1] School of Chemical Engineering and Advanced Materials, The University of Adelaide, Adelaide, SA 5005, Australia.

[2] Centre for Martials in Energy and Catalysis, The University of Adelaide, SA 5005, Australia.

[3] Department of Chemistry and Biochemistry & Advanced Materials and Liquid Crystal Institute, Kent State University, Kent, OH 44242, USA

* To whom correspondence should be addressed. Email: s.qiao@adelaide.edu.au (S-Z.Q.); yan.jiao@adelaide.edu.au (Y.J.)

This file contains:

- **Computational Details**
- **Supplementary Figures 1-6**
- **Supplementary Tables 1-8**
- **Supplementary References**

Computational Details

DFT Computation Parameters. Further to the computation details outlined in the manuscript, we used the plane-wave cutoff energy of 400 eV and the first order Methfessel-Paxton scheme with a smearing width of 0.2 eV. For static computations, the convergence criteria were 1×10^{-5} eV energy differences for solving the electronic wave function while geometry optimization were converged to within 2×10^{-2} eV/Å.

Interface Modelling. The water/Cu (100) interface was modeled with 32 explicit (four layers) water molecules on a three-layer (3×4×3) Cu(100) surface slabs. The bottom two layers of Cu atoms are fixed at their bulk positions (a=3.67 Å), also water molecules of the topmost layer are kept fixed to keep the water density of the interface close to the density of bulk water. All other atoms are allowed to relax during the simulation.

Molecular Dynamics. To describe the dynamic nature of hydrogen-bonding network, we carried out ab initio molecular dynamics (AIMD) simulation at 300 K and constructed the free energy profiles. A 1 fs time step with hydrogen mass set to 2 and the convergence criteria for electronic step set to 1×10^{-6} eV using the gamma point of the Brillouin zone. This system in canonical ensemble is first heated up from 0K to 300K by rescaling the velocities every 20 steps and then equilibrated at 300K for more than 30ps with a Nose-Hoover thermostat. Then adsorbates were added to the well-equilibrated interface; the initial configurations were determined by minima hopping.[1] The free energy profile for this reaction was obtained from thermodynamic integration of the potential of mean force along the reaction pathway produced either by 2ps AIMD simulations at 12 windows (NO dissociation) or slow-growth with a step size of 0.0008Å for most cases while for hard-to-converge cases, a smaller step size of 0.0005Å or 0.0002Å was used.[2] Both the Eley-Rideal (ER, water as proton source and Langmuir-Hinshelwood (LH, *H at the hollow site as the proton source) reaction mechanisms were considered for the proton coupled electron transfer step. The collective variables (CV) for elementary reactions are defined as the distance between hydrogen and carbon/nitrogen/oxygen atom of the reaction intermediate.

Determination of Electrode Potential

In the present work, we determine the electrode potential range versus SHE by referencing the work function of electrochemical interface to an experimental value of 4.44 eV for the standard hydrogen electrode (SHE).

$$U_{SHE} = (\phi - \phi_{SHE})/e = (\phi - 4.44)/e \quad (1)$$

where ϕ is the work function, which can be computed from DFT calculations. Similar schemes were also adopted for the study of electrochemical reduction of CO_2.[1, 2, 3, 4]

As an example of determining the electrode potential of the electrochemical interface, Figure S2 demonstrates that the work function varies with time along the AIMD trajectories. We found that although the temperature and potential energy converge quickly (Figure S1), it takes about 24ps for work functions to reach an acceptable equilibrium. The work function is reduced from the initial configuration until it reaches the equilibrium. However, computing work functions for each point of the trajectories requires significant computational resources. For other cases, we could not sample all the points and instead, only dozens of the last few picoseconds were considered. The work function values for Cu (100) with various intermediates are summarized in Table S1 and Table S2. These data show that although the adsorbed intermediates are different, the computed electrode potentials are in a narrow range (-0.85~-0.59 vs SHE), considering the errors of the methodology used for calculating the work function values (could up to 0.41 eV). Hence, we omitted calculations of the work functions for other intermediates with C-N bond like *CO-NO. As shown in the following section, the constant potential corrections are minor where the determination of the electrode potential of each intermediate is needed.

Constant Potential Corrections

All the calculations were done when the number of electrons was fixed, which meads the work function as well as the electrode potential referenced with standard hydrogen electrode changes along the reaction coordinates. However, the electrochemical measurements were conducted under fixed potential referenced with a certain reference electrode. To get electrochemical barrier at constant potential, we adopted methods developed by Chan and Nørskov based on a capacitor model:

$$\Delta E = \frac{\Delta q \bullet \Delta \phi}{2} \quad (2)$$

where ΔE, Δq, and $\Delta \phi$ corresponds to the energy correction due to change of electrode potential, charges and workfunctions.[5, 6] In this present work, we calculated the capacitors instead according to the following equation 3, which also proved valid.[4] Using the method proposed in Ref. 13, the calculated capacitance (C) is 1.27 e/V. When the electrode potential

values in Table S1 and S2 are assigned to the parameters in eq.3, we found that for most cases (only one exception), the energy correction term is less than 0.06 eV (corresponding to the work function change of 0.3eV), which is less than the standard deviation of the calculated barriers in Table S3-8. Hence, we concluded that the constant potential correction terms are minor in the present study.

$$\Delta E = \frac{C \bullet \Delta\phi^2}{2} \tag{3}$$

Supplementary Results

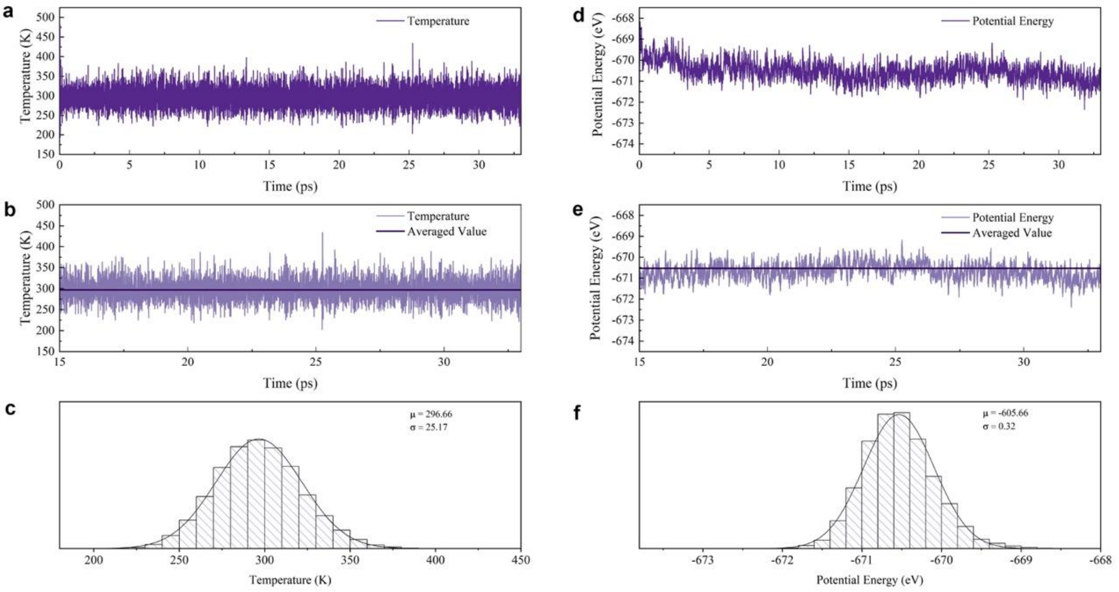

Supplementary Fig. 1. | Equilibrium of the system. Temperature and potential energies of the constructed electrochemical interface using *NO on Cu (100) at about -0.75V *vs.* standard hydrogen electrode (SHE) as an example. Temperature evolution along the (a) full timescale, (b) from 15 picosecond onward, and (c) distribution of temperature. Potential energies evolution along the (d) full timescale, (e) from 15 picoseconds onward, and (f) distribution of potential energies.

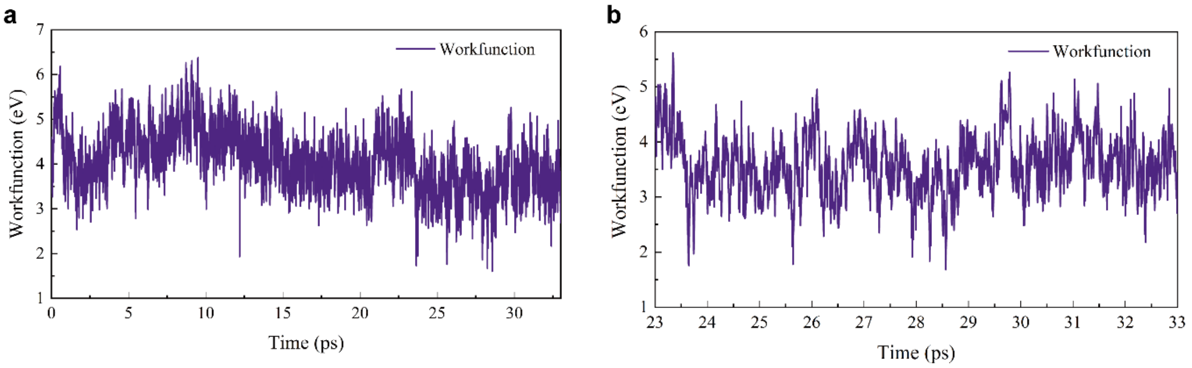

Supplementary Fig. 2. | Workfunction evolution during the simulation. Work function values along the trajectories. (a) entire 33 picoseconds; (b) the last 10 picoseconds.

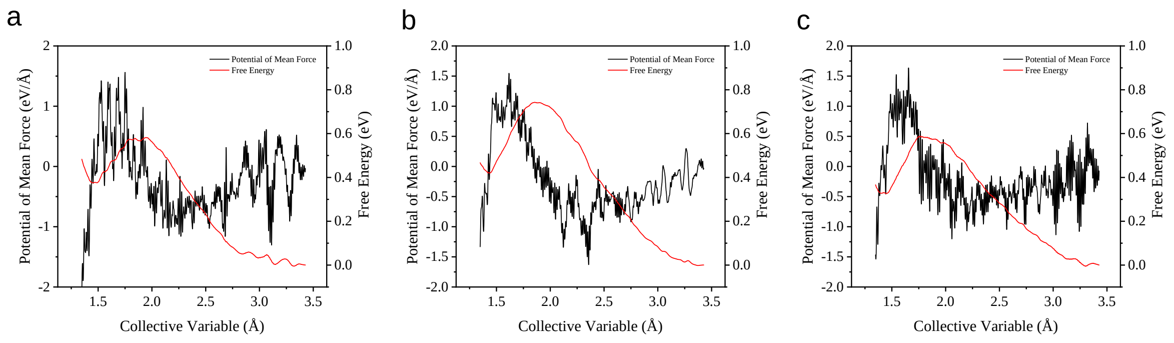

Supplementary Fig. 3. | Reaction barrier calculation for *NO+*CO→ *CO-NO. Potential of mean force derived from slow growth simulation (black line) and thermodynamic integrations (red line) of *NO+*CO→ *CO-NO at about -0.75V vs SHE with a step size of 0.0008Å. (a), (b) and (c) are three independent tasks.

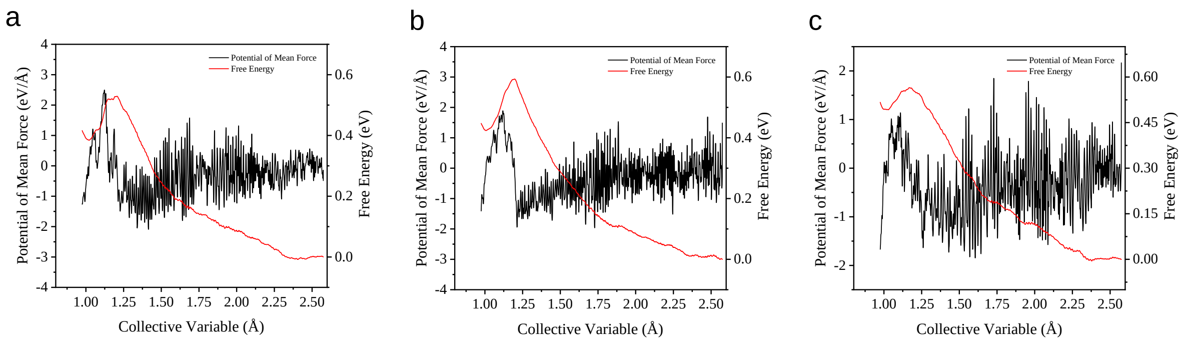

Supplementary Fig. 4. | Reaction barrier calculation for *NO + H_2O + e^-→ *NOH + OH^-. Potential of mean force derived from slow growth simulation (black line) and thermodynamic integrations (red line) of *NO + H_2O + e^-→ *NOH + OH^- at about -0.75V vs SHE with a step size of 0.0005Å. (a), (b) and (c) are three independent tasks.

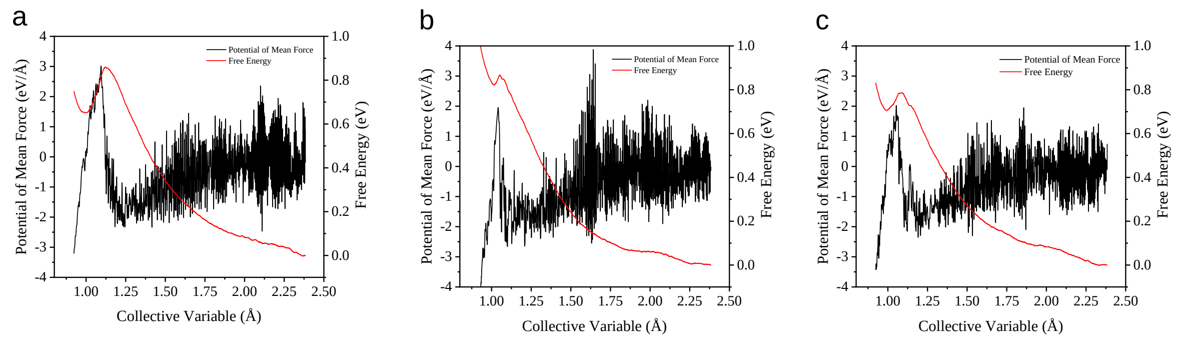

Supplementary Fig. 5. | Reaction barrier calculation for *HNO + H_2O + e^-→ *HNOH + OH^-. Potential of mean force derived from slow growth simulation (black line) and thermodynamic integrations (red line) of *HNO + H_2O + e^-→ *HNOH + OH^- at about -0.75V vs SHE with a step size of 0.0002Å. (a), (b) and (c) are three independent tasks.

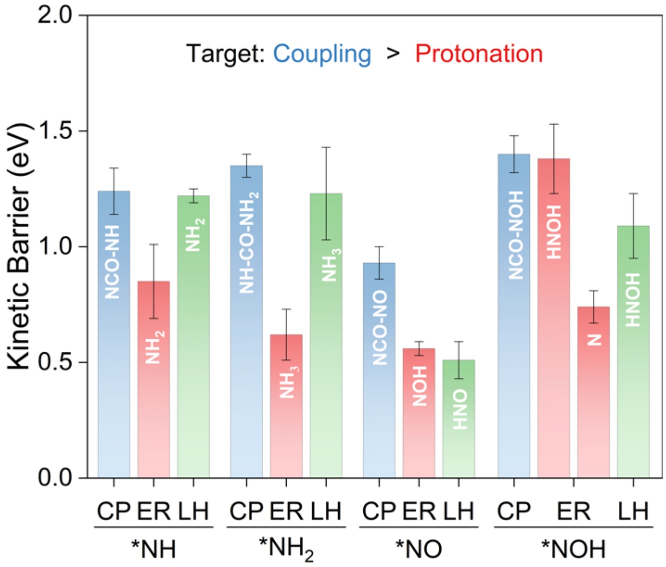

Supplementary Fig. 6. | The second C-N formation. Comparison of the coupling of various nitrogenous intermediates with *NCO and protonation.

Supplementary Table 1. Workfunctions of initial and transition state for various reduction step from NO^* to ammonia at ~ -0.75V *vs.* standard hydrogen electrode (SHE).

ID	Reaction Equation	**Initial State (eV)**	**Transition State (eV)**
1	$NO^* + H_2O + e^- \rightarrow NOH^* + OH^-$	3.74(±0.40)	3.99(±0.51)
2	$NO^* \rightarrow N^* + O^*$	3.74(±0.40)	N/A
3	$NO^* + H_2O + e^- \rightarrow HNO^* + OH^-$	3.74(±0.40)	3.92(±0.21)
4	$NO^* + H^* \rightarrow HNO^*$	3.74(±0.40)	3.73(±0.20)
5	$NOH^* \rightarrow N^* + OH^-$	3.77(±0.31)	3.82(±0.37)
6	$NOH^* + H_2O + e^- \rightarrow HNOH^* + OH^-$	3.77(±0.31)	3.81(±0.23)
7	$NOH^* + H^* \rightarrow HNOH^* + OH^-$	3.77(±0.31)	3.80(±0.29)
8	$HNO^* + H_2O + e^- \rightarrow HNOH^* + OH^-$	3.73(±0.31)	3.72(±0.40)
9	$HNO^* + H^* \rightarrow HNOH^*$	3.73(±0.31)	3.71(±0.37)
10	$HNO^* + H_2O + e^- \rightarrow ONH_2^* + OH^-$	3.73(±0.31)	3.77(±0.52)
11	$HNO^* + H_2O + e^- \rightarrow ONH_2^* + OH^-$	3.73(±0.31)	3.96(±0.50)
12	$N^* + H_2O + e^- \rightarrow NH^* + OH^-$	3.83(±0.28)	3.75(±0.39)
13	$HNOH^* \rightarrow NH^* + OH^-$	3.71(±0.30)	3.68(±0.39)
14	$HNOH^* + H^* \rightarrow NH_2OH^*$	3.71(±0.30)	4.22(±0.31)
15	$ONH_2^* + H_2O + e^- \rightarrow HONH_2^* + OH^-$	3.59(±0.41)	3.69(±0.38)
16	$ONH_2^* + H^* \rightarrow HONH_2^*$	3.59(±0.41)	3.76(±0.47)
17	$ONH_2^* + H_2O + e^- \rightarrow ONH_3^* + OH^-$	3.59(±0.41)	3.53(±0.36)
18	$ONH_2^* + H^* \rightarrow ONH_3^* + OH^-$	3.59(±0.41)	3.55(±0.44)

19	$NH^* + H_2O + e^- \rightarrow NH_2^* + OH^-$	3.68(±0.32)	3.78(±0.69)
20	$NH^* + H^* \rightarrow NH_2^*$	3.68(±0.32)	N/A
21	$NH_2^* + H_2O + e^- \rightarrow NH_3^* + OH^-$	3.85(±0.23)	3.76(±0.27)
22	$NH_2^* + H^* \rightarrow NH_3^*$	3.85(±0.23)	3.71(±0.40)

Supplementary Table 2. Workfunctions of initial and transition state for various reduction steps from NO* to ammonia at ~-1.5 V *vs.* standard hydrogen electrode (SHE).

ID	Reaction Equation	Initial State (eV)	Transition State(eV)
1	$NO^* + H_2O + e^- \rightarrow NOH^* + OH^-$	3.13(±0.56)	3.17(±0.42)
3	$NO^* + H_2O + e^- \rightarrow HNO^* + OH^-$	3.13(±0.56)	N/A
4	$NO^* + H^* \rightarrow HNO^*$	3.13(±0.56)	3.10(±0.48)
5	$NOH^* + H_2O + e^- \rightarrow N^* + OH^-$	2.88(±0.36)	3.14(±0.25)
6	$NOH^* + H_2O + e^- \rightarrow HNOH^* + OH^-$	2.88(±0.36)	3.17(±0.44)
7	$NOH^* + H^* \rightarrow HNOH^* + OH^-$	2.88(±0.36)	3.00(±0.48)
8	$N^* + H_2O + e^- \rightarrow NH^* + OH^-$	N/A	3.30(±0.42)
9	$N^* + H^* \rightarrow NH^*$	N/A	3.31(±0.32)
10	$NH^* + H_2O + e^- \rightarrow NH_2^* + OH^-$	N/A	3.11(±0.40)
11	$NH^* + H^* \rightarrow NH_2^*$	N/A	N/A
13	$NH_2^* + H_2O + e^- \rightarrow NH_3^* + OH^-$	2.78(±0.54)	2.91(±0.50)
14	$NH_2^* + H^* \rightarrow NH_3^*$	2.78(±0.54)	3.00(±0.50)

Supplementary Table 3. Free energy barriers for pathways from *NO to ammonia on Cu (100) at ~ -0.75V *vs.* standard hydrogen electrode (SHE).

ID	Reaction Equation	Reaction Barrier (eV)	CV	Step Size(Å)
1	$NO^{*}+H_2O+e^{-}\rightarrow NOH^{*}+OH^{-}$	0.56(±0.03)	r_{O-H}	0.0005
2	$NO^{*}\rightarrow N^{*}+O^{*}$	0.81(±0.04)	r_{N-O}	NA
3	$NO^{*}+H_2O+e^{-}\rightarrow HNO^{*}+OH^{-}$	1.75(±0.11)	r_{N-H}	0.0008
4	$NO^{*}+H^{*}\rightarrow HNO^{*}$	0.51(±0.08)	r_{N-H}	0.0008
5	$NOH^{*}\rightarrow N^{*}+OH^{-}$	0.74(±0.07)	r_{O-H}	0.0002
6	$NOH^{*}+H_2O+e^{-}\rightarrow HNOH^{*}+OH^{-}$	1.38(±0.15)	r_{N-H}	0.0008
7	$NOH^{*}+H^{*}\rightarrow HNOH^{*}$	1.09(±0.14)	r_{N-H}	0.0008
8	$HNO^{*}+H_2O+e^{-}\rightarrow HNOH^{*}+OH^{-}$	0.84(±0.04)	r_{O-H}	0.0002
9	$HNO^{*}+H^{*}\rightarrow HNOH^{*}$	1.36(±0.04)	r_{O-H}	0.0008
10	$HNO^{*}+H_2O+e^{-}\rightarrow ONH_2^{*}+OH^{-}$	0.47(±0.09)	r_{N-H}	0.0008
11	$HNO^{*}+H^{*}\rightarrow ONH_2^{*}$	1.25(±0.25)	r_{N-H}	0.0008
12	$N^{*}+H_2O+e^{-}\rightarrow NH^{*}+OH^{-}$	0.21(±0.09)	r_{N-H}	0.0008
13	$HNOH^{*}\rightarrow NH^{*}+OH^{-}$	0.50(±0.04)	r_{O-N}	0.0008
14	$HNOH^{*}+H_2O+e^{-}\rightarrow NH_2OH^{*}+OH^{-}$	1.15(±0.07)	r_{N-H}	0.0008
15	$ONH_2^{*}+H_2O+e^{-}\rightarrow HONH_2^{*}+OH^{-}$	0.41(±0.06)	r_{O-H}	0.0008
16	$ONH_2^{*}+H^{*}\rightarrow HONH_2^{*}$	1.33(±0.24)	r_{O-H}	0.0008
17	$ONH_2^{*}+H_2O+e^{-}\rightarrow ONH_3^{*}+OH^{-}$	1.34(±0.25)	r_{N-H}	0.0008
18	$ONH_2^{*}+H^{*}\rightarrow ONH_3^{*}+OH^{-}$	0.88(±0.15)	r_{N-H}	0.0008

19	$NH^* + H_2O + e^- \rightarrow NH_2^* + OH^-$	0.85(±0.16)	r_{N-H}	0.0008
20	$NH^* + H^* \rightarrow NH_2^*$	1.22(±0.03)	r_{N-H}	0.0008
21	$NH_2OH^* \rightarrow NH_2^* + OH^-$	0.67(±0.05)	r_{N-O}	0.0008
22	$NH_2^* + H_2O + e^- \rightarrow NH_3^* + OH^-$	0.62(±0.11)	r_{N-H}	0.0008
23	$NH_2^* + H^* \rightarrow NH_3^*$	1.23(±0.20)	r_{N-H}	0.0008

Supplementary Table 4. Free energy barriers for possible C-N bond formation steps on Cu (100) at ~ -0.75V *vs.* standard hydrogen electrode (SHE).

ID	Reaction Equation	Reaction Barrier(eV)	CV	Step Size(Å)
1	$NO^* + CO^* \rightarrow ON\text{-}CO^*$	0.64(±0.09)	r_{N-O}	0.0008
2	$NOH^* + CO^* \rightarrow HON\text{-}CO^*$	0.66(±0.14)	r_{N-O}	0.0008
3	$HNO^* + CO^* \rightarrow HNO\text{-}CO^*$	0.95(±0.11)	r_{N-O}	0.0008
4	$N^* + CO^* \rightarrow N\text{-}CO^*$	0.82(±0.08)	r_{N-O}	0.0008
5	$NH^* + CO^* \rightarrow HN\text{-}CO^*$	0.66(±0.09)	r_{N-O}	0.0008
6	$NH_2^* + CO^* \rightarrow NH_2\text{-}CO^*$	1.15(±0.14)	r_{N-O}	0.0008

Supplementary Table 5. Free energy barriers for protonation pathways after first C-N bond formation at ~ -0.75V *vs.* standard hydrogen electrode (SHE).

ID	Reaction Equation	Reaction Barrier(eV)	CV	Step Size(Å)
1	$NO\text{-}CO^{*} + H_2O + e^{-} \rightarrow CO-NOH^{*} + OH^{-}$	0.12(±0.07)	r_{O-H}	0.0002
2	$CO-NOH^{*} \rightarrow CO-N^{*} + OH^{-}$	0.36(±0.03)	r_{O-N}	0.0008
3	$N\text{-}CO^{*} + H_2O + e^{-} \rightarrow CO-NH^{*} + OH^{-}$	0.93(±0.10)	r_{N-H}	0.0005
4	$N\text{-}CO^{*} + H^{*} \rightarrow CO-NH^{*}$	1.16(±0.08)	r_{N-H}	0.0008
5	$NH\text{-}CO^{*} + H_2O + e^{-} \rightarrow CO-NH_2^{*} + OH^{-}$	0.87(±0.08)	r_{N-H}	0.0008
6	$NH\text{-}CO^{*} + H^{*} \rightarrow CO-NH_2^{*}$	1.42(±0.15)	r_{N-H}	0.0008

Supplementary Table 6. Free energy barriers for possible second C-N bond formation steps towards urea on Cu (100) at pH 7 and 300 K at -0.75 V vs SHE.

ID	Reaction Equation	Reaction Barrier(eV)	CV	Step Size(Å)
1	$N\text{-}CO^{*} + NO^{*} \rightarrow NCO-NO^{*}$	0.93(±0.07)	r_{C-N}	0.0008
2	$N\text{-}CO^{*} + NOH^{*} \rightarrow NCO-NOH^{*}$	1.40(±0.08)	r_{C-N}	0.0008
3	$N\text{-}CO^{*} + NH^{*} \rightarrow NCO-NH^{*}$	1.24(±0.10)	r_{C-N}	0.0008
4	$N\text{-}CO^{*} + NH_2^{*} \rightarrow NCO-NH_2^{*}$	1.35(±0.05)	r_{C-N}	0.0008
5	$NH\text{-}CO^{*} + NO^{*} \rightarrow NHCO-NO^{*}$	1.04(±0.12)	r_{C-N}	0.0008
6	$NH\text{-}CO^{*} + NOH^{*} \rightarrow NHCO-NOH^{*}$	0.82(±0.06)	r_{C-N}	0.0008
7	$NH\text{-}CO^{*} + NH^{*} \rightarrow NHCO-NH^{*}$	0.51(±0.08)	r_{C-N}	0.0008
8	$NH\text{-}CO^{*} + NH_2^{*} \rightarrow NHCO-NH_2^{*}$	1.68(±0.17)	r_{C-N}	0.0008

Supplementary Table 7. Free energy barriers for pathways after amide bond formation to urea on Cu (100) at pH 7 and 300 K at -0.75 V *vs.* standard hydrogen electrode (SHE).

ID	Reaction Equation	Reaction Barrier(eV)	CV	Step Size(Å)
1	$(NH)_2CO^* + H_2O + e^- \rightarrow NH_2CONH^* + OH^-$	0.52(±0.02)	r_{N-H}	0.0005
2	$(NH)_2CO^* + H^* \rightarrow NH_2CONH^*$	1.33(±0.11)	r_{N-H}	0.0008
3	$NH_2CONH^* + H_2O + e^- \rightarrow CO(NH_2)_2 + OH^-$	0.50(±0.08)	r_{N-H}	0.0008
4	$NH_2CONH^* + H^* \rightarrow CO(NH_2)_2$	1.27(±0.02)	r_{N-H}	0.0008

Supplementary Table 8. Pathway from *NO to NH_3 on Cu (100) at pH 7 and 300 K at ~ -1.5V *vs.* standard hydrogen electrode (SHE).

ID	Reaction Equation	Reaction Barrier(eV)	CV	Step Size(Å)
1	$NO^* + H_2O + e^- \rightarrow NOH^* + OH^-$	0.22(±0.01)	r_{N-H}	0.0005
2	$NO^* + H^* \rightarrow HNO^*$	0.61(±0.08)	r_{N-H}	0.0008
3	$NOH^* + H_2O + e^- \rightarrow N^* + OH^-$	0.24(±0.04)	r_{N-O}	0.0008
4	$NOH^* + H_2O + e^- \rightarrow HNOH^* + OH^-$	0.66(±0.03)	r_{N-H}	0.0008
5	$NOH^* + H^* \rightarrow HNOH^* + OH^-$	1.07(±0.10)	r_{N-H}	0.0008
6	$N^* + H_2O + e^- \rightarrow NH^* + OH^-$	0.22(±0.02)	r_{N-H}	0.0008
7	$N^* + H^* \rightarrow NH^*$	1.11(±0.03)	r_{N-H}	0.0008
8	$NH^* + H_2O + e^- \rightarrow NH_2^* + OH^-$	0.54(±0.01)	r_{N-H}	0.0008
9	$NH^* + H^* \rightarrow NH_2^*$	1.19(±0.03)	r_{N-H}	0.0008
10	$NH^* + CO^* \rightarrow NH - CO^*$	1.25(±0.04)	r_{N-C}	0.0008
11	$NH_2^* + H_2O + e^- \rightarrow NH_3^* + OH^-$	0.24(±0.09)	r_{N-H}	0.0008
12	$NH_2^* + H^* \rightarrow NH_3^*$	1.40(±0.07)	r_{N-H}	0.0008
13	$NH^* + CO_{(gas)} \rightarrow NH - CO^*$	0.62(±0.10)	r_{N-C}	0.0005

Supplementary References

1. Montoya JH, Shi C, Chan K, Norskov JK. Theoretical Insights into a CO Dimerization Mechanism in CO_2 Electroreduction. *J Phys Chem Lett* **6**, 2032-2037 (2015).

2. Cheng T, Xiao H, Goddard WA. Free-Energy Barriers and Reaction Mechanisms for the Electrochemical Reduction of CO on the Cu(100) Surface, Including Multiple Layers of Explicit Solvent at pH 0. *The Journal of Physical Chemistry Letters* **6**, 4767-4773 (2015).

3. Cheng T, Xiao H, Goddard WA, 3rd. Reaction Mechanisms for the Electrochemical Reduction of CO_2 to CO and Formate on the Cu(100) Surface at 298 K from Quantum Mechanics Free Energy Calculations with Explicit Water. *J Am Chem Soc* **138**, 13802-13805 (2016).

4. Cheng T, Xiao H, Goddard WA, 3rd. Full atomistic reaction mechanism with kinetics for CO reduction on Cu(100) from ab initio molecular dynamics free-energy calculations at 298 K. *Proc Natl Acad Sci USA* **114**, 1795-1800 (2017).

5. Chan K, Nørskov JK. Potential Dependence of Electrochemical Barriers from ab Initio Calculations. *J Phys Chem Lett* **7**, 1686-1690 (2016).

6. Chan KR, Norskov JK. Electrochemical Barriers Made Simple. *J Phys Chem Lett* **6**, 2663-2668 (2015).
